# Optimizing Soluble Cues for Salivary Gland Tissue Mimetics Using a Design of Experiments (DoE) Approach

**DOI:** 10.3390/cells11121962

**Published:** 2022-06-18

**Authors:** Lindsay R. Piraino, Danielle S. W. Benoit, Lisa A. DeLouise

**Affiliations:** 1 Department of Biomedical Engineering, University of Rochester, Rochester, NY 14627, USA; lpirain2@ur.rochester.edu (L.R.P.); danielle.benoit@rochester.edu (D.S.W.B.); 2 Department of Biomedical Genetics, University of Rochester Medical Center, Rochester, NY 14642, USA; 3 Department of Environmental Medicine, University of Rochester Medical Center, Rochester, NY 14642, USA; 4 Wilmot Cancer Institute, University of Rochester Medical Center, Rochester, NY 14642, USA; 5 Materials Science Program, University of Rochester, Rochester, NY 14627, USA; 6 Department of Chemical Engineering, University of Rochester, Rochester, NY 14627, USA; 7 Center for Musculoskeletal Research, University of Rochester Medical Center, Rochester, NY 14642, USA; 8 Department of Dermatology, University of Rochester Medical Center, Rochester, NY 14642, USA

**Keywords:** salivary gland, design of experiments, Mist1, acinar cell, EGFR inhibitor

## Abstract

The development of therapies to prevent or treat salivary gland dysfunction has been limited by a lack of functional in vitro models. Specifically, critical markers of salivary gland secretory phenotype downregulate rapidly ex vivo. Here, we utilize a salivary gland tissue chip model to conduct a design of experiments (DoE) approach to test combinations of seven soluble cues that were previously shown to maintain or improve salivary gland cell function. This approach uses statistical techniques to improve efficiency and accuracy of combinations of factors. The DoE-designed culture conditions improve markers of salivary gland function. Data show that the EGFR inhibitor, EKI-785, maintains relative mRNA expression of Mist1, a key acinar cell transcription factor, while FGF10 and neurturin promote mRNA expression of Aqp5 and Tmem16a, channel proteins involved in secretion. Mist1 mRNA expression correlates with increased secretory function, including calcium signaling and mucin (PAS-AB) staining. Overall, this study demonstrates that media conditions can be efficiently optimized to support secretory function in vitro using a DoE approach.

## 1. Introduction

Saliva is essential for oral health and disruption of normal salivary flow can lead to issues with eating, speaking, oral health, and tooth decay [1]. There are many causes of salivary gland hypofunction, including radiotherapy for head and neck cancer, Sjogren’s syndrome, various medications, and as a comorbidity of other diseases [1,2,3]. Radiotherapy is used to treat head and neck cancer for around 50,000 new patients in the US each year [4], and up to 80% of patients experience symptoms such as oral mucositis, loss of taste, dental caries, and damage to the periodontium and bone [5]. Sjogren’s syndrome is an autoimmune disease that is characterized by lymphocyte infiltration, cytokine production, and autoantibodies that affect the salivary and lacrimal glands [6]. Additionally, various medications, including antihistamines, antihypertensives, β-blockers, and antidepressants [3], as well as many conditions, including diabetes mellitus, Parkinson’s disease, cystic fibrosis, and human immunodeficiency virus (HIV) infection can lead to dry mouth [1,7].

Efforts to study the pathology of salivary gland hypofunction and develop therapies to prevent it have been hampered by a lack of functional salivary gland models. Salivary gland cells cultured in vitro rapidly lose their secretory phenotype and function [8,9]. Tissue engineering approaches have been utilized to identify function-promoting microenvironments through the use of hydrogels and other engineered extracellular matrices, and have had success in achieving high cell viability and proper localization of key structural proteins [10,11,12,13]. However, recapitulation of the acinar cell phenotype remains a major challenge in the field. Our previous study showed that cells could be cultured for up to 14 days in vitro, but expression of key markers such as Mist1 dropped significantly [13]. We hypothesized that optimization of the soluble cues could help address this limitation.

Several different types of media supplements (factors) have been tested to help improve function, including chemical inhibitors, growth factors, and neurotrophic factors [11,12,14,15,16,17,18,19]. However, several limitations to these studies exist: (1) factors were tested in isolation or one at a time, (2) factors were tested at only one concentration, or (3) direct effects on acinar cells were not investigated.

To address these limitations, a design of experiments (DoE) approach was used to optimize soluble cues for in vitro culture using a salivary gland tissue chip model [13]. A DoE is a statistical method to design and analyze experiments through systematic manipulation of variables to increase efficiency and improve predictions of outcomes. In comparison to methods that change one factor at a time and have the potential to miss important interactions between factors, DoEs allow multiple factors to be varied simultaneously while still permitting analysis of their individual effects to be separated using statistical analysis [20]. Despite their many advantages, DoEs have rarely been employed to study biological events and have not been applied specifically to salivary gland research.

The process used here consisted of a Plackett–Burman screening design to test seven different factors, followed by a Box–Behnken response surface design to optimize the concentrations of the top three factors revealed by the screen [20]. The effects were analyzed by measuring mRNA expression of three important acinar cell genes: Mist1, an acinar cell specific transcription factor necessary for the secretory phenotype [21]; Aqp5, a water channel protein involved in saliva secretion [22]; and Tmem16a, a calcium-activated chloride channel that contributes to chloride efflux during secretion [23]. Mist1 mRNA expression was increased by the addition of an EGFR inhibitor, EKI-785, while Aqp5 and Tmem16a mRNA expression were increased by a combination of FGF10 and neurturin. Since opposing results were obtained with Mist1 and Aqp5/Tmem16a mRNA expression, two media were created: M media containing EKI-785 to increase Mist1 mRNA expression and AT media containing FGF10 and neurturin to increase Aqp5 and Tmem16a mRNA expression. These media were tested for secretory function using a calcium signaling assay and periodic acid-Schiff’s reagent/Alcian Blue (PAS-AB) staining for mucins. Data show the M media improves calcium signaling and mucin staining compared to AT and base media and further improvements in relative mRNA expression can be made using a ROCK inhibitor for the first 24 h prior to M media (R24M).

## 2. Materials and Methods

### 2.1. Experimental Design

The objective of this study was to identify soluble cue(s) that prevent the precipitous loss of Mist1 expression that occurs when salivary gland cells are cultured in vitro. In particular, the aim was to test several factors that have been previously shown to impact secretory characteristics for their individual effects on Mist1, as well as to test the effects of combining factors. A DoE approach was designed in JMP Pro 15 (SAS) using seven factors (FGF10, neurturin, EGFR inhibitor, ROCK inhibitor, Apolipoprotein E, insulin, and TGFβR1 inhibitor) identified from the literature [12,16,17,18,24,25,26,27,28,29] and tested with a microbubble (MB)-hydrogel culture system [13].

### 2.2. Animals

Female SKH hairless mice, backcrossed six generations to C57BL/6J mice, at 6–12 weeks of age were used in this study. Only female mice were used due to known sex differences in rodent glands [30,31]. Animals were maintained on a 12 h light/dark cycle and group-housed with food and water available ad libitum. All procedures were approved and conducted in accordance with the University Committee on Animal Resources at the University of Rochester Medical Center (UCAR #2010-24E).

### 2.3. Primary Cell Isolation

Mice were euthanized with CO_2_ followed by cervical dislocation and the removal of the submandibular glands. The glands were dissociated with a razor blade for ~5 min, followed by enzymatic digestion with 50 U/mL collagenase type II (Gibco, Thermo Fisher Scientific, Waltham, MA, USA, 17101-015) and 1 mg/mL hyaluronidase (Sigma Aldrich, St. Louis, MO, USA, H3506) in Hank’s buffered salt solution (HBSS) with 15 mM HEPES at 37 °C for 30 min. Dispersed SMG clusters were subsequently passed through 100 and 20 μm filters to isolate clusters between 20–100 µm. The digestion protocol produces cell cluster sizes evenly distributed between 20 to 100 µm [32]. The isolated clusters were re-suspended in base culture medium, defined below.

### 2.4. Microbubble (MB) Array Fabrication

Microbubble (MB) arrays were fabricated in poly(dimethyl) siloxane (PDMS) using gas expansion molding as previously described [33]. A 10:1 ratio (by weight) of base to curing agent (Dow Corning Sylgard 184 PDMS, GMID: 04019862) was mixed thoroughly and poured over a silicon wafer template. The template contained deep etched cylindrical pits with a 200 µm diameter, spaced 600 µm apart. Casts were cured at 100 °C for 2 h, then peeled off the template. This process results in the formation of near-spherical cavities over each of the cylindrical pits. Chips were punched from the template using a circular punch (0.7 cm diameter) and glued into 48-well plates using PDMS (5:1 weight% ratio of base to curing agent). Chips with ~100 MBs were primed in a desktop vacuum chamber with 70% ethanol for sterilization and washed with PBS overnight prior to cell seeding.

### 2.5. Cell Seeding

Isolated submandibular gland cell clusters were encapsulated in poly (ethylene glycol) (PEG) hydrogels within MB arrays as previously described [13]. Briefly, the cells were resuspended in hydrogel precursor solution containing 2 mM norbornene-functionalized 4-arm 20 kDa PEG-amine macromers, 4 mM of the dicysteine functionalized MMP-degradable peptide (GKKCGPQG↓IWGQCKKG), 0.05 weight% of the photoinitiator lithium phenyl-2,4,6-trimethylbenzoylphosphinate (LAP) [34], and 0.1 mg/mL laminin (Gibco, 23017-015) in PBS [35]. The PEG-amine, MMP-degradable peptide, and LAP were fabricated in-house as previously described [13,34]. The cell/gel precursor solution (25 µL) was pipetted onto the microbubble chips and incubated for 30 min at 37 °C. Cell clusters we seeded into the MBs by gravity. To maximize cell seeding, the cells were redispersed by pipetting every 10 min during the seeding period, allowing more clusters to fall into each MB. The hydrogels were then polymerized using a Hand-Foot 1000 A broad spectrum UV light with a UVC filter for 1.5 min and cultured with 0.5 mL of media that was changed every 2 days. Seeding the cluster suspension in MB array is a statistical process, with multiple clusters deposited into individual MBs. These clusters aggregate and within 2–3 days form SGm that continue to grow over time [13]. Heterogeneity in SGm size and/or cell composition may impact characteristics of individual SGm, but the results are averaged across the large array format.

### 2.6. Media

Base media consisted of a 1:1 ratio of Dulbecco’s Modified Eagle medium (DMEM, Gibco, 11995-065):Ham’s F-12 Nutrient Mixture (Corning, 10-080-CV) supplemented with 100 U/mL Penicillin and 100 μg/mL Streptomycin (Gibco, 15140-122), 2 mM Glutamine (Gibco, 35050-061), 0.5× N2 supplement (Gibco, 17502-048), 2.6 ng/mL insulin (Gibco, 12585-014), 2 nM dexamethasone (Sigma Aldrich, St. Louis, MO, USA, D4902), 20 ng/mL epidermal growth factor (EGF, Gibco, PHG0313), and 20 ng/mL basic fibroblast growth factor (bFGF, Gibco, PHG0021). Additional supplements that were added (at the concentration indicated in Table 1) during the DoE optimization included EGFR inhibitor EKI-785 (Sigma 233100), FGF10 (Invitrogen PHG0204), ROCK inhibitor Y-27632 (Tocris Bioscience 12-541), TGFβR1 inhibitor SB525334 (Sigma S8822), Apolipoprotein E (R & D Systems 4144-AE), neurturin (Sigma SRP3124), and insulin (Gibco 12585-014). Note that insulin is included in the base media at a low level, but its concentration was increased for the DoE.

### 2.7. Cell Viability

Cells were cultured for 7 days with media containing each DoE factor separately at the (+1) concentrations listed in Table 1. Cell viability was analyzed on day 7 by staining with calcein AM (4 µM, LIVE stain) and propidium iodide (4 µM, DEAD stain) in culture media for 30 min. Chips were rinsed once with PBS and imaged at 10× using an Olympus IX70 microscope with FITC (Excitation 488 nm/Emission 525 nm) and Texas Red (Excitation 595 nm/Emission 620 nm) for LIVE and DEAD stains, respectively. Overlay images between the two fluorescence channels were created in ImageJ. Brightfield images were used to determine sphere diameter using the line measurement tool in ImageJ. Two perpendicular lines were drawn across the sphere and measured using the scale set in ImageJ (Figure S1C). The average of these lines was calculated for each sphere for one chip (~50 MBs).

### 2.8. Plackett–Burman Design

Guided by the literature [36], a 24-run folded-over Plackett–Burman design (Table 2) was used to identify factors with a significant effect on Mist1, Aqp5, and Tmem16a mRNA expression to eliminate factors with negligible or negative effects. Folding over the design avoids obscuring the main effects of the factors with two-factor interactions. Seven factors were tested at two concentrations, with the high level selected from the literature (Table 1). Cells were cultured for seven days, with media changed every two days, and the media was supplemented with the combinations defined by each run. Relative mRNA expression was measured and the results were fitted in JMP Pro 15 (SAS), first using stepwise regression with minimum Akaike Information Criteria (AIC) to reduce number of terms in the model to fit the number of degrees of freedom. It was then fitted to a first-order polynomial using standard least squares.

### 2.9. Box–Behnken Design

The concentrations of top three factors chosen from the Plackett–Burman design (EGFR inhibitor, FGF10, neurturin) were optimized using a 15-run Box-Behnken design (Table 3). Factors were tested at three equidistant levels (Table 1), guided by the Plackett–Burman results and the literature [29,37], for their effects on relative mRNA expression of Mist1, Aqp5, and Tmem16a. Cells were cultured for seven days, with the media supplemented with the additives defined by each run and the media was changed every two days. The entire design was duplicated, and mRNA expression results were averaged before analysis in JMP Pro 15 (SAS). Results were fitted to a second-order polynomial using standard least squares.

### 2.10. RNA Extraction and qPCR

After 2, 7, or 14 days of culture, chips were placed in an Eppendorf tube containing 400 µL TRK lysis buffer (Omega Bio-tek, Norcross, GA, USA, HCR003) with 8 µL β-mercaptoethanol (EMD Millipore, Burlington, MA, USA, 444203) and stored at −80 °C. For RNA extraction, chips were thawed and vortexed vigorously to dislodge the cell contents out of the MBs. The solution was transferred to homogenizer tubes (Omega Bio-tek, Norcross, GA, USA, HCR003) and extracted using the E.Z.N.A Total RNA Kit I (Omega Bio-tek, Norcross, GA, USA, R6834). RNA was quantified with a spectrophotometer (NanoDrop Lite) and transcribed to cDNA with the iScript™ cDNA synthesis kit (Bio-Rad, Hercules, CA, USA, 170-8890) according to the manufacturer’s instructions. qPCR was performed using PowerUp™ SYBR™ Green Master Mix (Applied Biosystems, A25742) with a CFX Connect™ Real-Time System (Bio-Rad, Hercules, CA, USA) using 3 technical replicates per sample. Results were normalized to housekeeping gene Rps29 and day 0 freshly isolated cells and analyzed using the 2^−ΔΔCt^ method. Primer sequences are shown in Supplementary Table S1.

### 2.11. Calcium Signaling

An in-chip calcium signaling assay was conducted as previously described [13]. Briefly, cells were loaded with the calcium-sensitive dye Calbryte 520 AM (AATBioquest, Sunnyvale, CA, USA, 20650) at 10 µM in imaging buffer (137 mM NaCl, 4.7 mM KCl, 1 mM Na_2_HPO_4_, 1.26 mM CaCl_2_, 0.56 mM MgCl_2_, 5.5 mM glucose, 10 mM HEPES) with 0.04% Pluronic F-127 for 1 h at 37 °C. The dye was washed out and then the chips were imaged using a Cytation 5 Multi-Mode Reader with an Injection Module (Bio-tek, Norcross, GA, USA). Fluorescent (Excitation/Emission = 490/525 nm) time lapse images were taken once per second for 30 s, an agonist was injected using the automated injector, and chips imaged for another 150 s. Chips were first stimulated with carbachol (final concentration = 1 µM) for the entire 3 min data collection period and then washed twice with imaging buffer and reused for calcium signaling analysis with ATP as an agonist (final concentration = 40 µM).

For data analysis, images were opened as a stack in ImageJ to create a time-lapse video. Regions of interest (ROIs) were defined by thresholding on highly fluorescent regions at 35 s, 70 s, and 140 s in the time course and merging images to create a composite ROI map. A custom ImageJ macro was used to measure the integrated density of each ROI over time. Data were processed to remove non-responsive ROIs and plotted as mean fluorescence intensity normalized to the baseline intensity (F/F0) over time. Traces are the averaged responses of *n* = 3 experiments, plotted similarly to others in the literature [38,39,40]. Latency was calculated as the time it took for the signal to reach 4 standard deviations above the baseline. Duration was calculated as the amount of time the signal stayed over 4 standard deviations above the baseline signal.

### 2.12. Periodic Acid-Schiff’s Reagent-Alcian Blue (PAS-AB) Staining

Chips were pretreated with 25 U/mL diastase for 20 min to remove glycogen, then washed with deionized water and stained with 1% Alcian blue (Electron Microscopy Sciences, pH 2.5, Electron Microscopy Sciences, 26026-13, Hatfield, PA, USA) for 30 min. Chips were rinsed extensively and stained with 0.5% periodic acid (Sigma Aldrich, St. Louis, MO, USA, P7875) for 2 min followed with Schiff’s reagent (Sigma Aldrich, St. Louis, MO, USA) for 15 min. A final rinse with tap water was performed before imaging with a Nikon Eclipse E800 microscope equipped with a Spot Insight 12 MP CMOS camera [13]. PAS-AB staining was quantified by measuring the area of stain corresponding with the hue of freshly isolated salivary gland cells divided by the total area of stained cells.

### 2.13. Statistical Analysis

Designs and analyses for the Plackett–Burman and Box–Behnken DoEs were generated using JMP Pro 15 (SAS). Statistics were determined using standard least squares for linear regression (Figures 3 and 5) and one-way ANOVA for overall model *p*-values (Supplementary Tables S3 and S6) with Benjamini–Hochberg post-hoc test to determine the effects of individual factors (Supplementary Tables S4 and S7). 3D plots for Figure 6, Supplementary Figures S3 and S4 were generated using MATLAB R2020b. Graphs for Figures 2–5 and 7–10 were generated using Prism 9 (GraphPad, San Diego, CA, USA). Two-way ANOVA with Sidak’s post-hoc test (Figures 7, 8 and 10) was determined using Prism 9. One-way ANOVA with Tukey’s multiple comparisons was used for Supplementary Figures S8 and S9. Pseudocolored images were created in ImageJ. Number of experiments (*n*) is shown in each figure caption; each experiment consisted of one array with ~50 MBs. Statistics for Figure 10, Figures S8 and S9 were calculated using the number of MBs.

## 3. Results

### 3.1. Individual Factors Are Non-Toxic

**Figure 1:**
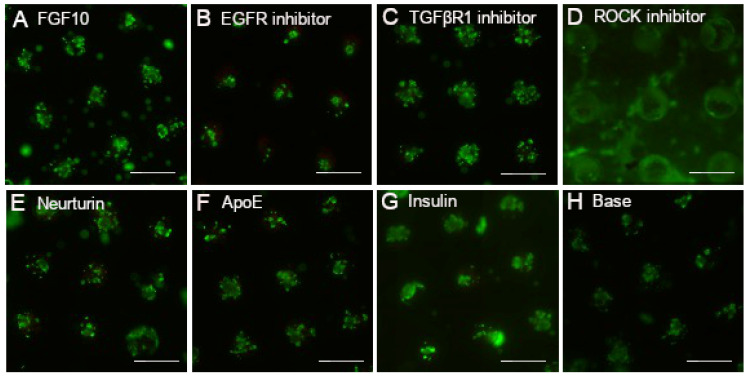
DoE factors are non-toxic to SGm. Cell viability staining using calcein AM (green) for live cells and propidium iodide (red) for dead cells for the individual factors used in the Plackett–Burman DoE: FGF10 (**A**), EGFR inhibitor (**B**), TGFβR1 inhibitor (**C**), ROCK inhibitor (**D**), neurturin (**E**), apolipoprotein E (**F**), and insulin (**G**). Base media is shown for comparison (**H**). Scale bar is 400 µm. Concentrations used are shown in Table 1.

Seven factors were chosen from the literature based on their reported influence in supporting secretory characteristics: FGF10, EGFR inhibitor EKI-785, ROCK inhibitor Y-27632, TGFβR1 inhibitor SB525334, neurturin, apolipoprotein E, and insulin [11,12,16,18,26,27,28]. These factors were tested individually at concentrations determined from literature (Table 1) for cell viability using calcein AM/propidium iodide staining after 7 days of culture (Figure 1). Most of the tissue mimetics in the array displayed high viability with negligible propidium iodide-positive dead cells. Notably, ROCK inhibitor Y-27632 induced morphological changes, as evidenced by the loss of sphere structure (Figure 1D) and outgrowth onto the chip surface (Supplementary Figure S1A). Additionally, EGFR inhibitor EKI-785 produced smaller spheres (Figure 1B) compared to control media (Figure 1H), while minimal morphological changes were observed with the other factors (Supplementary Figure S1B).

### 3.2. FGF10, EGFR Inhibitor, ROCK Inhibitor, and Neurturin Increase Acinar Cell Relative mRNA Expression

**Figure 2:**
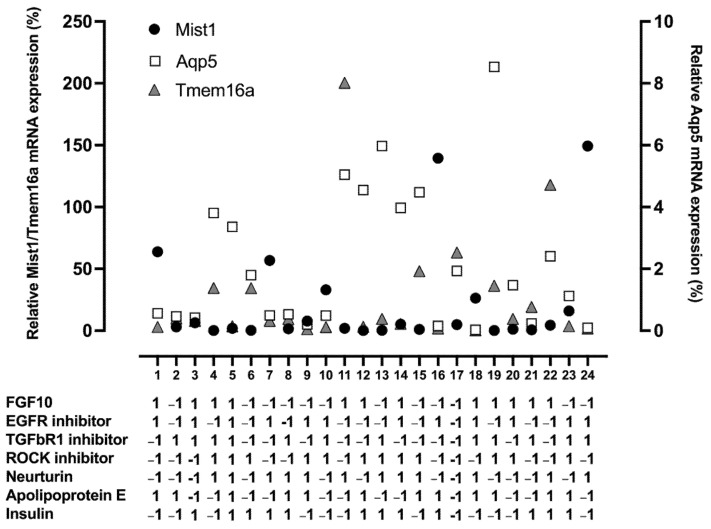
Individual runs of the Plackett–Burman show changes in relative mRNA expression. Scatter plot of the relative mRNA expression of the three responses, Mist1 (black circles), Aqp5 (white squares), and Tmem16a (gray triangles), for the Plackett–Burman screen. Each tick on the x-axis corresponds to the run number, with the levels (−1 or 1) of each factor shown underneath. mRNA expression is relative to the housekeeping gene Rps29 and day 0 tissue. Relative Aqp5 mRNA expression is plotted on the right y-axis and relative Mist1 and Tmem16a mRNA expression are plotted on the left y-axis.

**Figure 3:**
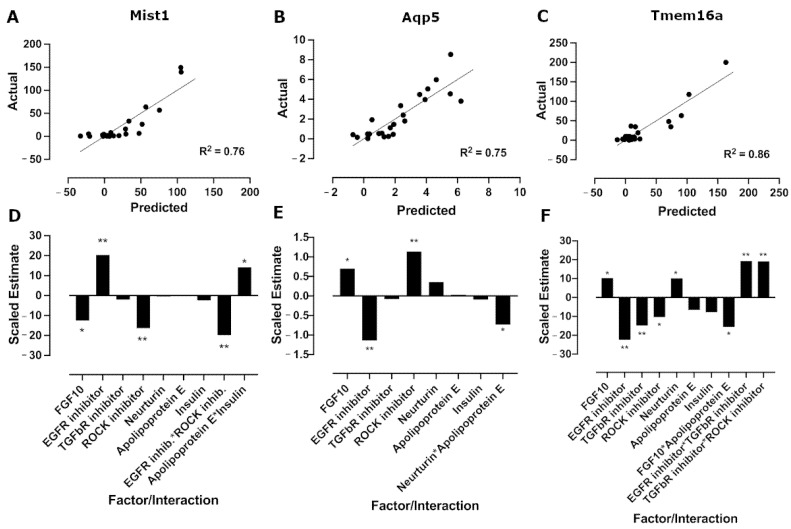
EGFR inhibitor, FGF10, neurturin, and ROCK inhibitor increase relative mRNA expression. Scatter plot of the predicted (model) values against the actual (experimental) mRNA expression with best fit line and R^2^ values for Mist1 (**A**), Aqp5 (**B**), and Tmem16a (**C**) for the Plackett–Burman DoE. Linear regression was used to determine the best fit line and R^2^ values. Main effects plots showing the scaled estimates of factors and significant interactions for Mist1 (**D**), Aqp5 (**E**), and Tmem16a (**F**). *p*-values were determined using one-way ANOVA with Benjamini–Hochberg post-hoc test. * *p* < 0.05; ** *p* < 0.01.

A folded-over Plackett–Burman DoE was used to screen for factors that result in an increase in relative mRNA expression of key acinar markers, Mist1, Aqp5, and Tmem16a. Results of the individual runs for all three responses are shown in Figure 2. These data are also reported in Supplementary Table S2. These results were then analyzed separately for each response using linear regression, and the model/predicted value, calculated using the fitted equation (Supplementary Table S3), was plotted against the actual/experimental mRNA expression (Figure 3A–C) to give R^2^ values of 0.76, 0.75, and 0.86 and *p*-values of 0.004, 0.002, and 0.0004 for Mist1, Aqp5, and Tmem16a, respectively (Supplementary Table S3).

Analysis of the main effects of individual factors (Figure 3D–F) showed that the EGFR inhibitor had a positive effect on Mist1 mRNA expression, while FGF10 and the ROCK inhibitor had negative effects. For Aqp5, FGF10 and the ROCK inhibitor increased relative mRNA expression, while the EGFR inhibitor reduced mRNA expression. FGF10 and neurturin increased Tmem16a relative mRNA expression, while the EGFR, TGFβR1, and ROCK inhibitors downregulated Tmem16a mRNA expression. There were also a few interactions with significant effects, but these did not outweigh the main effects of the individual factors. The *p*-values for individual factors and significant interactions are shown in Supplementary Table S4.

Based on these results, there were four factors with positive effects on at least one acinar cell marker: EGFR inhibitor, FGF10, ROCK inhibitor, and neurturin. Despite a positive effect on Aqp5 mRNA expression, the ROCK inhibitor was removed from further analysis due to its significant negative effects on both Mist1 and Tmem16a. Furthermore, the ROCK inhibitor caused excessive proliferation and outgrowth from the MBs (Supplementary Figure S1A). Therefore, the EGFR inhibitor, FGF10, and neurturin were used in a Box–Behnken DoE for optimizing factor concentrations in a three-level design.

### 3.3. The EGFR Inhibitor Promotes Mist1 mRNA Expression While FGF10 and Neurturin Promote Aqp5 and Tmem16a mRNA Expression

**Figure 4:**
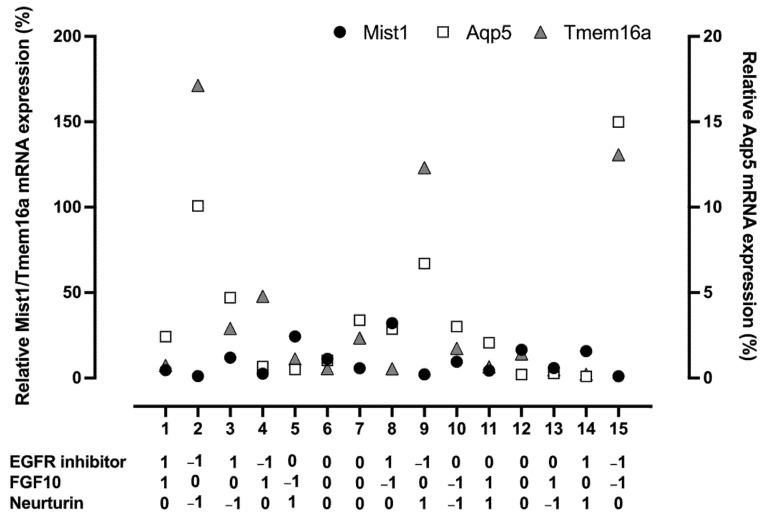
Individual runs from Box–Behnken show an impact on relative mRNA expression. Scatter plot of the results of the Box–Behnken runs for relative mRNA expression of Mist1 (black circles), Aqp5 (white squares), and Tmem16a (gray triangles). Each tick on the x-axis corresponds to the run number, with the levels (−1 and 1) of each factor shown underneath. mRNA expression is relative to the housekeeping gene Rps29 and day 0 tissue. Relative Mist1 and Tmem16a mRNA expression are plotted on the left y-axis and relative Aqp5 mRNA expression is plotted on the right y-axis.

A duplicated Box–Behnken DoE was used to optimize the concentrations of the EGFR inhibitor, FGF10, and neurturin using three levels (Table 1). The concentrations for FGF10 and neurturin were increased to 500 ng/mL and 10 ng/mL, respectively, for the third level (+1) to see if higher concentrations would boost the positive effects observed in the screen. The (+1) level for the EGFR inhibitor was unchanged due to the reduced sphere size and increased cytotoxicity observed at higher EGFR inhibitor concentrations (Supplementary Figure S2). Results for individual runs are shown in Figure 4 and in tabular format in Supplementary Table S5.

**Figure 5:**
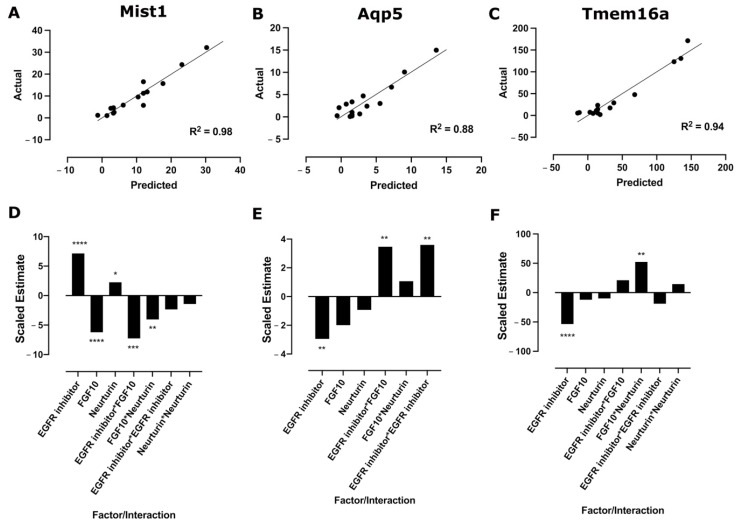
The EGFR inhibitor has strong positive effect on Mist1 while FGF10 and neurturin improve relative Aqp5 and Tmem16a mRNA expression. Scatter plot of the predicted (model) values against the actual (experimental) values with best fit line and R^2^ values for Mist1 (**A**), Aqp5 (**B**), and Tmem16a (**C**) for the Box–Behnken DoE. Linear regression was used to determine the best fit line and R^2^ values. Main effects plots showing the scaled estimates of factors and significant interactions for Mist1 (**D**), Aqp5 (**E**), and Tmem16a (**F**). *p*-values were determined using one-way ANOVA with Benjamini–Hochberg post-hoc test. * *p* < 0.05; ** *p* < 0.01; *** *p* < 0.001; **** *p* < 0.0001.

Results were fitted to a second-order polynomial for each response (Supplementary Table S6), which was used to generate predicted values that were plotted against actual data (Figure 5A–C), giving R^2^ values of 0.98, 0.88, and 0.94 and *p*-values of <0.0001, 0.003, and 0.001 for Mist1, Aqp5, and Tmem16a mRNA expression, respectively (Supplementary Table S6). Analysis of the main effects revealed that the EGFR inhibitor once again had a positive effect on Mist1 mRNA expression, with the greatest effect at the highest concentration (0.5 µM) and a negative effect on both Aqp5 and Tmem16a mRNA expression (Figure 5D–F; Supplementary Table S7). Neurturin had a small but significant effect on Mist1 mRNA expression that was not present in the Plackett–Burman. This is likely due to the 10-fold increase in concentration tested for neurturin for the Box–Behnken (1 ng/mL for Plackett–Burman; 10 ng/mL for Box–Behnken). Additionally, increased concentrations of FGF10 and neurturin had negative effects on Aqp5 and Tmem16a mRNA expression. The EGFR inhibitor and FGF10 had a strong interaction for Aqp5; however, it did not outweigh the negative effects of the individual factors. The interaction between FGF10 and neurturin was positive for both Aqp5 and Tmem16a, although it was not significant for Aqp5.

**Figure 6:**
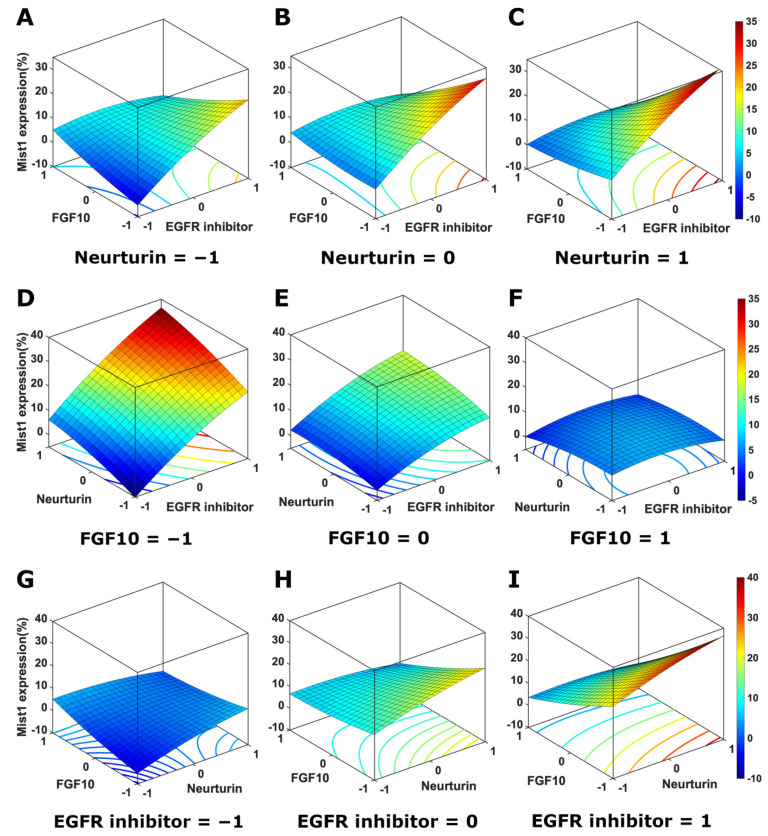
The EGFR inhibitor and FGF10 have opposing effects on Mist1 mRNA expression. 3D surface response and contour plots for the Box–Behnken DoE for Mist1 mRNA expression as a function of the concentration levels of EGFR inhibitor and FGF10 (**A**–**C**) with neurturin fixed at level −1 (**A**), 0 (**B**), and 1 (**C**); EGFR inhibitor and neurturin (**D**–**F**) with FGF10 at level −1 (**D**), 0 (**E**), and 1 (**F**); and neurturin and FGF10 (**G**–**I**) with the EGFR inhibitor at level −1 (**G**), 0 (**H**), and 1 (**I**). Concentrations levels correspond to values in Table 1.

Three-dimensional response surface plots and contour plots for the Box–Behnken DoE show the effects of the three factors on Mist1 mRNA expression (Figure 6), Aqp5 (Supplementary Figure S3), and Tmem16a (Supplementary Figure S4). For Mist1, decreasing FGF10 and increasing EGFR inhibitor concentration had large effects on Mist1 mRNA expression, while increasing the neurturin concentration resulted in a small increase in Mist1 mRNA expression (Figure 6). For Aqp5 and Tmem16a, the highest mRNA expression levels were predicted at the lowest concentration (0 ng/mL) for all soluble cues.

### 3.4. Model Validation Confirms Increases in Acinar Cell mRNA Expression in Mist1-Optimized and Aqp5/Tmem16a-Optimized Media

**Figure 7:**
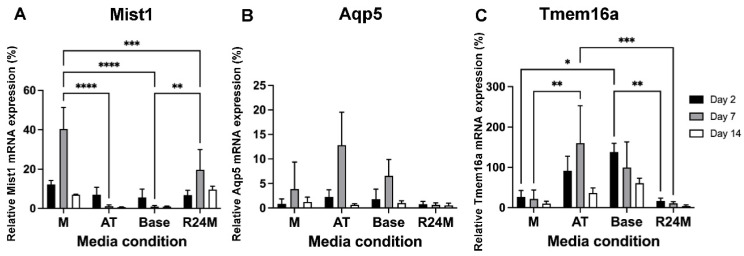
Model validation confirms increases in acinar cell mRNA expression in Mist1-optimized and Aqp5/Tmem16a-optimized media. Relative mRNA expression of Mist1 (**A**), Aqp5 (**B**), and Tmem16a (**C**) under different media conditions, Mist1-optimized (M), Aqp5/Tmem16a-optimized (AT), base (no additives), and ROCK inhibitor for 24 h, following by M (R24M) media after culturing for 2 (black), 7 (gray), and 14 (white) days. Relative mRNA expression is normalized to housekeeping gene Rps29 and day 0 tissue. Data are represented as mean ± SD, *n* = 3. Statistics were calculated using two-way ANOVA with Sidak’s post-hoc test. * *p* < 0.05, ** *p* < 0.01, *** *p* < 0.001, **** *p* < 0.0001.

Due to opposing effects of the EGFR inhibitor and FGF10, two optimized media were developed: one containing 0.5 µM EGFR inhibitor to optimize Mist1 mRNA expression (M media) and one containing 1 ng/mL neurturin and 100 ng/mL FGF10 to optimize Aqp5 and Tmem16a mRNA expression (AT media). Relative mRNA expression of Mist1, Aqp5, and Tmem16a was measured and compared to base media. As expected, an increase in Mist1 mRNA expression was observed in M media compared to base media, while AT media increased Aqp5 and Tmem16a mRNA expression (Figure 7). Mist1 mRNA expression was retained at ~40% of day 0 levels after 7 days of culture, compared to the predicted value of 24%. Aqp5 mRNA expression was measured at 12.8%, compared to the predicted 12.6%, and Tmem16a was measured at 160%, compared to the predicted 151%.

This trend continues out to 14 days for Mist1, with 7-fold higher Mist1 mRNA expression for M media compared to base media (Figure 7A). For Aqp5, there was no significant difference between any of the media conditions (Figure 7B), while Tmem16a mRNA expression was the highest in base media after 14 days, although the data are not statistically significant (Figure 7C). These results indicate that while Mist1 mRNA expression is enhanced at 7 days, longer-term maintenance at 14 days could still be improved.

### 3.5. Addition of a ROCK Inhibitor for the First 24 h Had Minimal Impact on Mist1 mRNA Expression, but Decreased mRNA Expression of Duct and Myoepithelial Markers

The ROCK inhibitor, Y27632, has been used to promote cell viability and decrease cell stress following cell isolation [24,41,42,43]. However, it has also been reported that the ROCK inhibitor causes a loss of 3D architecture and cell spreading [17,44], which aligns with the aberrant cell growth observed here (Supplementary Figure S1). Thus, to utilize the benefits of the ROCK inhibitor, it was added to the media for the first 24 h, then switched to M media for the remainder of the culture period (R24M media) (Figure 7A–C). Mist1 mRNA expression was lower in R24M media (20%) than M media (40%) at day 7, but was slightly higher in R24M media (10% vs. 7%) at day 14 (Figure 7A). Both Aqp5 and Tmem16a mRNA expression were lower in R24M media compared to base media (Figure 7B,C).

**Figure 8:**
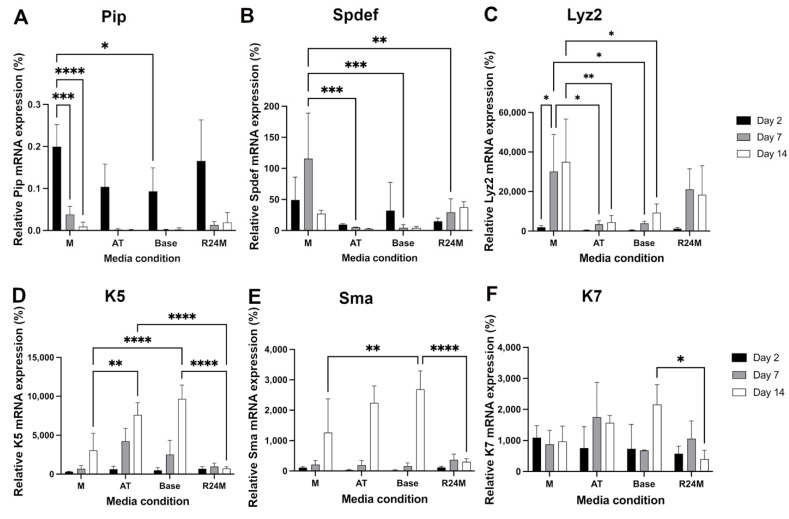
M and R24M media promote acinar cell genes and decrease duct/myoepithelial cell overgrowth. Relative mRNA expression of Pip (**A**), Spdef (**B**), Lyz2 (**C**), K5 (**D**), Sma (**E**), and K7 (**F**) under M, AT, Base, and R24M media conditions at day 2 (black), day 7 (gray), and day 14 (white). Relative mRNA expression is normalized to housekeeping gene Rps29 and day 0. Data are represented as mean ± SD, *n* = 3. Statistics were calculated using two-way ANOVA with Sidak’s post-hoc test. * *p* < 0.05, ** *p* < 0.01, *** *p* < 0.001, **** *p* < 0.0001.

Relative mRNA expression of other acinar cell markers, Pip, Spdef, and Lyz2, showed higher levels in M and R24M compared to other media, especially at day 14 (Figure 8A–C). As previously reported [13], base media conditions result in high mRNA expression of duct and myoepithelial cell markers (K5, K7, Sma, which is likely due to cell stress [45,46]); the AT media showed a similar behavior (Figure 8D–F). In contrast, both M and R24M showed lower mRNA expression of K5 and Sma, with considerably lower mRNA expression of both markers at day 14 in R24M compared to M media (Figure 8D,E). These data suggest that addition of the ROCK inhibitor for the initial period following cell isolation promotes acinar cell markers and hinders duct and myoepithelial markers with lasting effects out to 14 days of culture.

### 3.6. Mist1-Promoting Media, Alone and in Combination with ROCK Inhibition for 24 h, Showed an Enhanced Calcium Signaling Response to Carbachol

**Figure 9:**
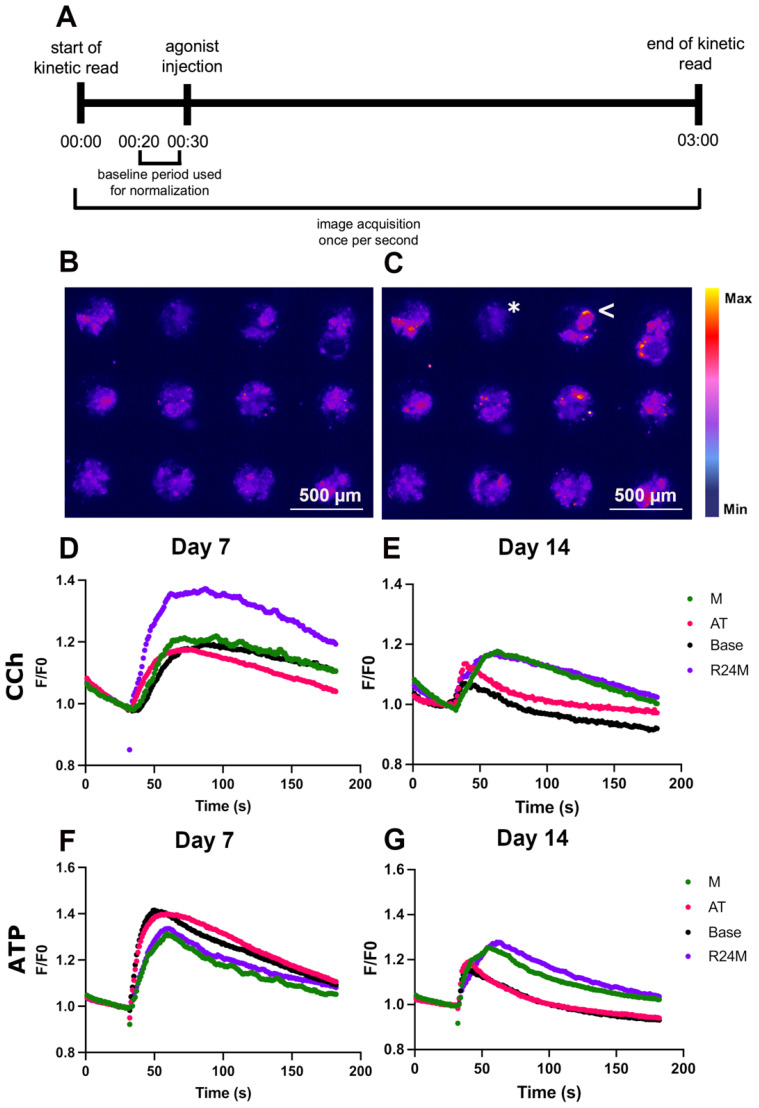
M and R24M media maintain calcium signaling. Timeline of the calcium signaling assay (**A**). Example images of the calcium signaling response before (**B**) and after (**C**) stimulation (example shown is day 7 AT stimulated with ATP). The asterisk (*) points to a non-responsive SGm and the less than sign (<) points to a responsive SGm. Time courses of the average response to CCh at day 7 (**D**) and day 14 (**E**) and ATP at day 7 (**F**) and day 14 (**G**). CCh (1 µM) or ATP (40 µM) were injected at 30 s. Data are represented as the average fluorescence intensity at each time point (*n* = 3) normalized to the baseline period (average fluorescence of time 20–29 s).

Calcium signaling is an essential process for driving saliva secretion [47]. Carbachol (CCh), a muscarinic agonist, and ATP, a purinergic agonist, both stimulate intracellular calcium flux. CCh helps drive fluid secretion, and increased response to CCh correlates with increased saliva production [48]. On the other hand, excessive purinergic signaling can be an indicator of cell stress or damage [49]. To detect calcium signaling, cells are loaded with the calcium-sensitive dye Calbryte 520 AM and then undergo a kinetic assay in which fluorescence images are taken at 1 s intervals for 30 s before injecting the agonist for the remainder of the 3-min image collection phase (Figure 9A). Example images show the cell response prior to (Figure 9B) and during stimulation (Figure 9C). At day 7, R24M showed the greatest response to CCh while the rest of the conditions were approximately equal (Figure 9D) and all media conditions showed about the same response to ATP (Figure 9F). At day 14, both CCh and ATP stimulation produced a higher calcium flux in M and R24M media (Figure 9E,G), approximately equivalent to the response produced at day 7, indicating that the cells maintain function over longer culture periods in these media conditions. While the same number of responsive ROIs was present at day 7 in all media conditions, there were more responsive ROIs for M and R24M media under both CCh and ATP stimulation at day 14 (Supplementary Figure S5A,B).

Small distinct regions responded to the agonists in M and R24M media, as compared to a globalized nonspecific response across the entire tissue mimetic for base and AT media (Supplementary Figure S6). Further, M and R24M showed fluctuations in calcium signaling in response to stimulation, as indicated by the oscillatory pattern of the averaged traces (Figure 9D–G), which is accentuated in the traces from individual ROIs with M media (Supplementary Figure S6A,C) compared to base media (Supplementary Figure S6B,D). Additionally, the ATP- and CCh-responsive regions overlapped in normal and AT media, while there were some distinct regions responding to only one agonist and some overlapping regions responding to both agonists in M and R24M media (Supplementary Figure S7), indicating spatially distinct heterogeneous cell populations exist under M and R24M. Minimal differences were seen in duration (Supplementary Figure S8) and latency (Supplementary Figure S9) at day 7 for both agonists, but M and R24M had longer duration and latency at day 14.

### 3.7. Periodic Acid-Schiff’s Reagent/Alcian Blue (PAS-AB) Staining Showed Preservation of Mucin Expression in M and R24M Media

**Figure 10:**
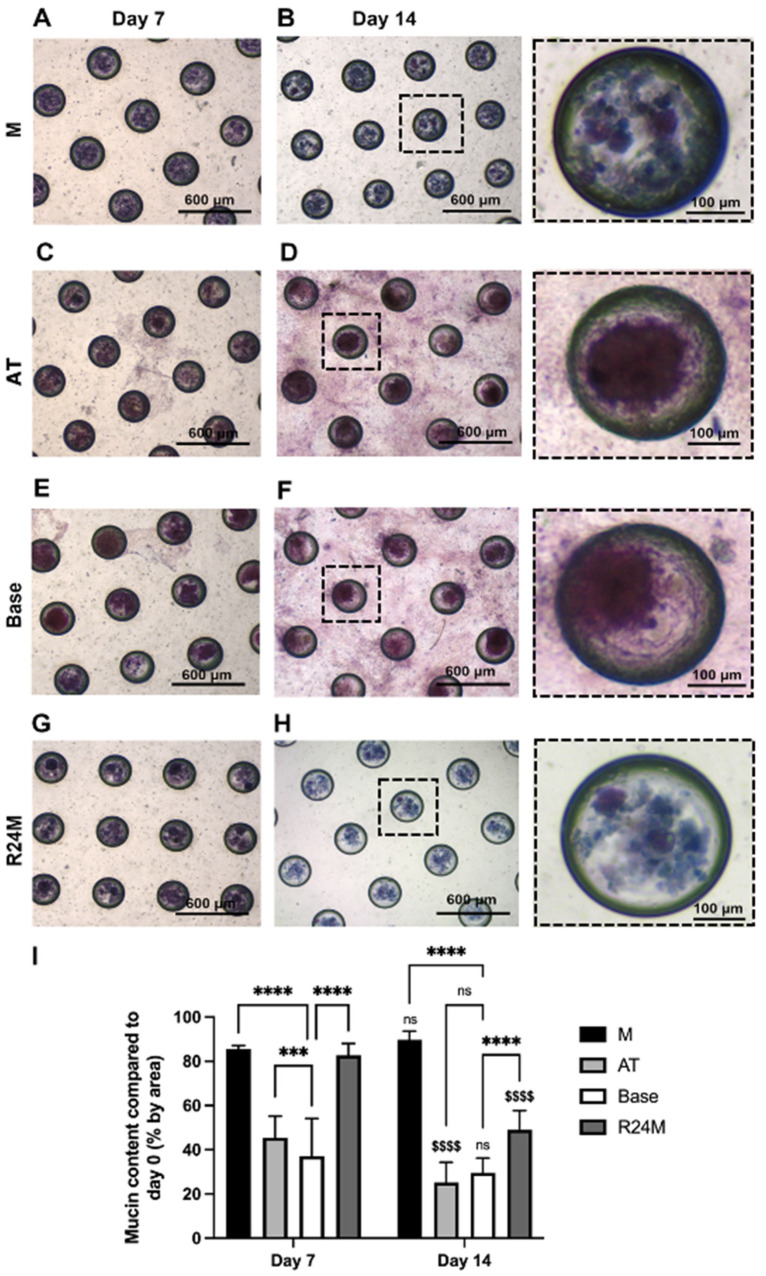
Mucin staining is preserved in M and R24M media. PAS-AB staining for M (**A**,**B**), AT (**C**,**D**), base (**E**,**F**), and R24M (**G**,**H**) media at day 7 and day 14, respectively. Quantification for the percent mucin content by area compared to day 0 tissue (**I**). Data are represented as mean ± SD, *n* = 3. Statistics were calculated using two-way ANOVA with Sidak’s post-hoc test. Brackets with asterisks compare between media conditions on the same day: ns = nonsignificant, *** *p* < 0.001, **** *p* < 0.0001. Money signs on day 14 data compare between time points for the same media condition: ns = nonsignificant, $$$$ *p* < 0.0001. Boxed MBs in (**B**,**D**,**F**,**H**) correspond with the magnified images to the right.

Mucins are glycoproteins that contribute to the viscoelasticity of saliva and line the mucosal surfaces to provide lubrication and antimicrobial properties [50]. Salivary gland tissue mimetics were cultured for 7 or 14 days in different media conditions and stained for mucins using periodic acid-Schiff’s reagent-Alcian Blue (PAS-AB). Submandibular glands are expected to stain for both PAS (pink) and AB (blue), creating a purple color [51]. M and R24M show preservation of freshly isolated salivary gland mucin expression (Supplementary Figure S10) at both 7 and 14 days (Figure 10A,B,G,H), with similar staining to in vivo tissue [52]. However, tissue mimetics cultured in base and AT media lose expression of the AB stain at both time points and show cell outgrowth from the MBs onto the chip surface at day 14, appearing as pink staining on the surface of the chip (Figure 10C–F). Overall, these images and quantification (Figure 10I) suggest that M and R24M preserve mucin expression in contrast to base or AT media.

## 4. Discussion

Here, media used to culture SGm were optimized for key acinar cell markers using a sequential design of experiments (DoE) approach. This approach consisted of a 24-run folded-over Plackett–Burman design to screen factors for increases in acinar cell mRNA expression, followed by a 15-run Box–Behnken design that tested the top three factors at three concentrations. Plackett–Burman designs are optimal for determining the main effects of many factors with a reduced number of runs. Folding over the design allowed for the determination of two-factor interactions to ensure that interactions did not obscure main effects, also known as aliasing. This was a critical feature of the sequential approach, as aliasing can lead to incorrect interpretation of the data and affect decisions regarding further optimization.

Results of the Plackett–Burman DoE showed that 0.5 µM EGFR inhibitor, 100 ng/mL FGF10, and 1 ng/mL neurturin had positive effects on at least one of the acinar cell genes measured, with the ROCK inhibitor removed from further testing due to concerns regarding the excessive proliferation and promotion of duct/myoepithelial cells. Results of the Box–Behnken DoE showed that 0.5 µM EGFR inhibitor with 10 ng/mL neurturin provided the optimal conditions for increasing Mist1 mRNA expression (Figure 6). Given its relatively small impact on Mist1 mRNA expression and its high cost, neurturin was removed from the optimized Mist1 media. For Aqp5 and Tmem16a, Box–Behnken results predicted the highest mRNA expression levels at 0 ng/mL for all factors. However, given the benefit for 100 ng/mL FGF10 and 1 ng/mL neurturin when directly tested in the Plackett–Burman DoE (Figure 2 and Figure 3), these additives and concentrations were used for the optimized Aqp5/Tmem16a media. Discrepancies between Plackett–Burman and Box–Behnken results are likely due to the wide concentration range tested in the Box–Behnken design (increased from 100 to 500 ng/mL for FGF10 and 1 to 10 ng/mL for neurturin), making it difficult to predict the optimal concentrations precisely. This highlights one benefit of the sequential DoE approach: data from both prediction models can be used to define optimized media more precisely.

DoEs are gaining in popularity for the optimization of media cues [36,53,54,55]. A major appeal of this approach is that optimization is achieved in far fewer experiments than would otherwise be possible, saving time and resources. For comparison, a full-factorial design would require 128 runs for seven factors at two levels or 2187 runs for seven factors at three levels, compared to the 39 total runs used here. A non-DoE approach might entail testing factors one at a time and combining positive factors either at the end or as they are identified; however, this method does not take into consideration the potential for interactions and thus the optimal solution may be missed.

Using the results of the joint DoE approach, two media were defined: one optimized for Mist1 mRNA expression (M media) with 0.5 µM EGFR inhibitor and the other optimized for Aqp5/Tmem16a mRNA expression (AT media) with 100 ng/mL FGF10 and 1 ng/mL neurturin. Media were directly tested and data supported the predictions. Compared to base media, Mist1 mRNA expression was increased by ~40-fold after 7 days in culture using M media (0.5 µM EGFR inhibitor). Retaining Mist1 expression in vitro has been a major challenge; addressing this issue is a significant contribution of this work. Using AT media (100 ng/mL FGF10, 1 ng/mL neurturin), Aqp5 mRNA expression was doubled and Tmem16a mRNA expression was increased by 60% at day 7. By day 14, relative mRNA expression of all markers had dropped in all media conditions, suggesting that further work could be done to improve longevity of the cultures. A fourth media condition was also investigated, consisting of a ROCK inhibitor for 24 h, followed by 0.5 µM EGFR inhibitor (R24M), to help reduce cell isolation-induced stress [24]. Little improvement was observed between R24M and M media for Mist1, Aqp5, and Tmem16a mRNA expression.

To further investigate the cell types present under different media conditions, relative mRNA expression of Pip, Spdef, Lyz2, K5, Sma, and K7 was analyzed. Higher mRNA expression of acinar cell-related genes (Pip, Spdef, Lyz2) and lower mRNA expression of duct/myoepithelial cell genes (K5, Sma, K7) were observed under M and R24M media, especially at days 7 and 14, indicating the preservation of more acinar-like cells in the EGFR inhibitor-containing media. In addition, R24M medium maintained much lower mRNA expression of K5 and Sma at day 14 compared to M medium, suggesting further benefits of the initial ROCK inhibitor addition. One possible explanation is that reduced cell stress following isolation from the ROCK inhibitor may diminish cell plasticity. Finally, calcium signaling and mucin (PAS-AB) staining showed that M and R24M media exhibit better preservation of secretory function compared to base and AT media, especially at day 14. Taken together, these results suggest that maintenance of Mist1 mRNA expression using soluble factors is more closely linked to other acinar cell markers and the secretory phenotype than Aqp5 and Tmem16a.

Similar to the work presented here, EGFR inhibitor, AG1478, has been reported to retain epithelial cells, but on the contrary, these cells were largely AQP5-positive [11]. One potential reason for this difference is that embryonic cells were used, in which expression patterns of Aqp5 are different than in adult tissue [22]. Additionally, EGFR plays an important role in branching morphogenesis, especially for ductal cell differentiation, so inhibiting it during the embryonic stage may result in aberrant development [56]. Other reports suggest that different EGFR inhibitors do not affect K5, K19, or Kit levels [12] or inhibit proliferation of K5 and K19 cells [57]. K5, K19, and Kit are duct/progenitor cell markers, so it is consistent that their decrease corresponds with an increase in the mature acinar cell marker, Mist1.

Neurturin has been shown to stimulate branching, innervation, and self-aggregation of spheres when combined with mesenchyme and parasympathetic ganglion, with a corresponding increase in AQP5 staining [12]. This corroborates our finding that neurturin can increase Aqp5 mRNA expression when combined with FGF10, which is produced by the mesenchyme. However, it was also reported that replacing the mesenchyme with media supplementation was not sufficient to establish branching morphogenesis, although AQP5 expression was not directly analyzed [12].

Similarly, FGF10 has been shown to increase AQP5, but in contrast to data from the DoE, an increase in Mist1 was also reported [19]. This could be explained by some notable differences between the studies. First, cells were cultured on 2D surfaces and passaged prior to single cell dispersion and seeding in Matrigel, whereas here, some tissue structure is retained during digestion and cells are immediately seeded within a 3D matrix. Second, 2D-cultured cells were all positive for K19 (a duct marker) prior to transferring to Matrigel^®^, whereas the cells used here were a heterogeneous mixture of acinar, duct, and myoepithelial cells [13]. Third, FGF10 was not added to the media until after 1 day of culture, while here, FGF10 was supplemented for the entirety of the culture period.

One perplexing outcome of the DoE was the observed inverse relationship between Mist1 and Aqp5/Tmem16a mRNA expression. Factors that promote Mist1 mRNA expression negatively impacted Aqp5 and Tmem16a and vice versa. While all three markers are present in acinar cells and important for proper acinar function, Aqp5 and Tmem16a are also expressed in intercalated ducts [22,23], so it is possible that increases in these markers reflect increases in the intercalated duct population rather than acinar cells. Additionally, it has previously been shown that stress induced by culturing salivary gland cells in vitro can cause cells of non-acinar lineage to express AQP5, while some cells of acinar lineage lose expression of AQP5 [45]. This type of cellular plasticity, also termed acinar-to-ductal metaplasia (ADM), has been studied extensively in the pancreas and is associated with a loss in Mist1 expression [58,59]. Importantly, activation of EGFR is known to be involved in ADM [60,61], while EGFR inhibition has previously been shown to prevent this process [25]. Another possible explanation is the presence of acinar cell subpopulations defined by expression of Smgc or Bpifa2 [62]. Future work could include mRNA expression of these markers to determine if different acinar subpopulations are promoted by the EGFR inhibitor, FGF10, and neurturin. Additionally, combining soluble cues with matrix optimization efforts is an important next step.

## 5. Conclusions

In conclusion, a long-standing issue of reduced Mist1 expression in vitro was addressed using a DoE approach to optimize culture media. Our results showed that an EGFR inhibitor increased Mist1 mRNA expression, while FGF10 and neurturin increased Aqp5 and Tmem16a mRNA expression after 7 days of culture. The beneficial effects of media supplementation are less pronounced by 14 days. Further analysis of media conditions revealed that EGFR inhibitor addition improved other acinar cell genes (Pip, Lyz2, Spdef) and indicators of secretory function (calcium signaling, mucin expression), while reducing duct and myoepithelial cell mRNA expression (K5, K7, Sma). Further reduction in overexpression of duct/myoepithelial cell genes at day 14 could be addressed by adding a ROCK inhibitor to the media for the initial 24 h of culture. This optimization will be useful for improving the relevance of in vitro salivary gland models, while the DoE approach can be adapted to address optimization efforts for improving other aspects of cell culture.

## Figures and Tables

**Table 1:** Factor Concentrations for the Plackett–Burman and Box–Behnken DoEs.

Plackett–Burman			
Factor	Low Level (−1)	High Level (+1)	Reference
Neurturin	0	1 ng/mL	[12]
TGFβR1 inhibitor	0	1 µM	[16,26]
EGFR inhibitor	0	0.5 µM	[11,25]
ROCK inhibitor	0	10 µM	[17,18,24]
Apolipoprotein E	0	1 µg/mL	[28]
FGF10	0	0.1 µg/mL	[19,29]
Insulin	0.03 µg/mL	10 µg/mL	[27]
**Box–Behnken**			
**Factor**	**Low Level (−1)**	**Middle Level (0)**	**High Level (+1)**
Neurturin	0	5 ng/mL	10 ng/mL
EGFR inhibitor	0	0.25 µM	0.5 µM
FGF10	0	0.25 µg/mL	0.5 µg/mL

**Table 2:** Experimental Design for the Plackett–Burman DoE Created using JMP. Each Row (Run) Indicates an Independent Experiment with the Factors Present At the −1 or +1 Level as Indicated. Concentrations for These Levels are Shown in Table 1.

	Factor						
Run #	Fgf10	EGFR Inhibitor	TGFβR1 Inhibitor	ROCK Inhibitor	Neurturin	Apolipoprotein E	Insulin
**1**	1	1	−1	−1	−1	1	−1
**2**	−1	−1	1	−1	−1	1	−1
**3**	1	1	1	−1	−1	−1	1
**4**	1	−1	1	1	1	−1	−1
**5**	1	1	1	1	1	1	1
**6**	−1	−1	−1	1	−1	−1	1
**7**	−1	1	−1	−1	1	−1	1
**8**	−1	−1	1	−1	1	1	1
**9**	−1	1	−1	1	1	1	−1
**10**	−1	1	1	1	−1	−1	−1
**11**	1	−1	−1	−1	1	−1	−1
**12**	1	−1	−1	1	−1	1	1
**13**	−1	−1	1	1	1	−1	1
**14**	1	1	−1	1	1	−1	1
**15**	−1	−1	−1	1	1	1	−1
**16**	−1	1	−1	−1	−1	1	1
**17**	−1	−1	−1	−1	−1	−1	−1
**18**	1	1	1	−1	1	1	−1
**19**	1	−1	1	1	−1	1	−1
**20**	1	1	−1	1	−1	−1	−1
**21**	1	−1	1	−1	−1	−1	1
**22**	1	−1	−1	−1	1	1	1
**23**	−1	1	1	1	−1	1	1
**24**	−1	1	1	−1	1	−1	−1

**Table 3:** Experimental Design for the Box–Behnken DoE Created using JMP. Each Row (Run) Indicates an Independent Experiment with the Factors Present At the −1, 0, or +1 Level as Indicated. Concentrations for These Levels are Shown in Table 1.

	Factor		
Run #	EGFR Inhibitor	FGF10	Neurturin
**1**	1	1	0
**2**	−1	0	−1
**3**	1	0	−1
**4**	−1	1	0
**5**	0	−1	1
**6**	0	0	0
**7**	0	0	0
**8**	1	−1	0
**9**	−1	0	1
**10**	0	−1	−1
**11**	0	1	1
**12**	0	0	0
**13**	0	1	−1
**14**	1	0	1
**15**	−1	−1	0

## Data Availability

The data presented in this study are available on request from the corresponding author.

## Supplementary Materials

The following supporting information can be downloaded at: https://www.mdpi.com/article/10.3390/cells11121962/s1; Figure S1: Morphological changes are induced by different media supplements; Figure S2: Higher concentrations of EGFR inhibitor show increased cytotoxicity and reduced sphere size; Figure S3: Optimal Aqp5 mRNA expression is predicted at low concentrations of FGF10 and neurturin; Figure S4: Optimal Tmem16a mRNA expression is predicted at low concentrations of FGF10 and neurturin; Figure S5: M and R24M media show increased number of responsive ROIs at day 14; Figure S6: Calcium signaling traces of individual ROIs showing oscillatory behavior in M media; Figure S7: Distinct regions respond to CCh vs. ATP in M media; Figure S8: M and R24M media show increased duration of the calcium signaling response at day 14; Figure S9: M and R24M media show longer latency of the calcium signaling response at day 14; Figure S10: Freshly isolated salivary gland cells in MBs show expression of both acidic and neutral mucins using PAS-AB staining, Table S1: Forward and reverse primer sequences for the genes used in qPCR; Table S2: Relative mRNA expression results for Mist1, Aqp5, and Tmem16a for individual runs of the folded-over Plackett–Burman DoE; Table S3: Model metrics (R^2^, R^2^ adjusted, model *p*-value) and model fit equation for the Plackett–Burman DoE for each of the three responses; Table S4: *p*-values for the individual factors and significant interactions for the Plackett–Burman DoE; Table S5: Relative mRNA expression results for Mist1, Aqp5, and Tmem16a for individual runs of the Box–Behnken DoE; Table S6: Model metrics (R^2^, R^2^ adjusted, model *p*-value) and model fit equation for the Box–Behnken DoE for each of the three responses; Table S7: *p*-values for the individual factors and interactions for the Box–Behnken DoE [63,64,65,66].
