# Supplementary material for: Optimizing Soluble Cues for Salivary Gland Tissue Mimetics Using a Design of Experiments (DoE) Approach
Source: Cells. 2022 Jun 18;11(12):1962. doi: 10.3390/cells11121962 (PMC9222211; doi:10.3390/cells11121962)
Supplement: Supplementary file 1 [file cells-11-01962-s001.zip › cells-1754910-supplementary.pdf]

Optimizing Soluble Cues for Salivary Gland Tissue Mimetics Using a Design of Experiments (DoE) Approach

Lindsay R. Piraino, Danielle S. W. Benoit and Lisa A. DeLouise

Table S1. Forward and reverse primer sequences for the genes used in qPCR.

Gene	Forward Primer	Reverse Primer	Ref
Rps29	AATACGGGCTGAACATGTGC	AGCATGATCGGTTCCACTTG	[13]
Mist1	GCTGACCGCCACCATACTTAC	TGTGTAGAGTAGCGTTGCAGG	[63]
Aqp5	GTGAGTGGTGGCCACATCAATCC	GGGAGTCCGTGGAGGAGAAGAT	[63]
Tmem16a	GACCTGGGCTATGAGGTTCA	GGCTGATGTCTTTGGGGATA	[64]
Pip	GGGTCTCTCATTCACATTCAGTG	TGATCTCCTGATTTTCCTGTGCT	[13]
Spdef	AAGGCAGCATCAGGAGCAATG	CTGTCAATGACGGGACACTG	[65]
Lyz2	ATGGAATGGCTGGCTACTATGG	ACCAGTATCGGCTATTGATCTGA	[66]
K7	CAGGCAGAGATTGACACCTT	GCGCCAGCTTGGTGTTCAG	[13]
K5	TCCAGTGTGTCCTTCCGAAGT	TGCCTCCGCCAGAACTGTA	[63]
Sma	GGACGTACAACTGGTATTGTGC	TCGGCAGTAGTCACGAAGGA	[63]

Table S2. Relative mRNA expression results for Mist1, Aqp5, and Tmem16a for individual runs of the folded-over Plackett-Burman DoE.

	Factors							Response		
**Run #**	**Fgf10**	**EGFR inhib.**	**TgfbR1 inhib.**	**ROCK inhib.**	**Ntrn**	**ApoE**	**Insulin**	**Mist1 (%)**	**Aqp5 (%)**	**Tmem16a (%)**
**1**	1	1	-1	-1	-1	1	-1	63.8	0.56	3.07
**2**	-1	-1	1	-1	-1	1	-1	3.05	0.46	9.99
**3**	1	1	1	-1	-1	-1	1	6.47	0.42	7.88
**4**	1	-1	1	1	1	-1	-1	0.22	3.81	34.57
**5**	1	1	1	1	1	1	1	1.97	3.36	3.52
**6**	-1	-1	-1	1	-1	-1	1	0.34	1.80	34.45
**7**	-1	1	-1	-1	1	-1	1	56.9	0.49	7.98
**8**	-1	-1	1	-1	1	1	1	1.55	0.53	9.38
**9**	-1	1	-1	1	1	1	-1	7.79	0.20	1.20
**10**	-1	1	1	1	-1	-1	-1	33.0	0.49	2.89
**11**	1	-1	-1	-1	1	-1	-1	1.97	5.05	200.38
**12**	1	-1	-1	1	-1	1	1	0.14	4.55	3.21
**13**	-1	-1	1	1	1	-1	1	0.29	5.97	9.60
**14**	1	1	-1	1	1	-1	1	5.23	3.97	5.55
**15**	-1	-1	-1	1	1	1	-1	1.04	4.48	48.18
**16**	-1	1	-1	-1	-1	1	1	139.5	0.15	1.87
**17**	-1	-1	-1	-1	-1	-1	-1	4.89	1.93	63.21
**18**	1	1	1	-1	1	1	-1	26.3	0.03	0.27
**19**	1	-1	1	1	-1	1	-1	0.25	8.53	36.37
**20**	1	1	-1	1	-1	-1	-1	1.20	1.47	9.55
**21**	1	-1	1	-1	-1	-1	1	0.68	0.24	19.14
**22**	1	-1	-1	-1	1	1	1	4.49	2.41	117.93
**23**	-1	1	1	1	-1	1	1	15.91	1.12	3.68
**24**	-1	1	1	-1	1	-1	-1	149.3	0.09	1.79

Table S3. Model metrics (R^2, R^2 adjusted, model p-value) and model fit equation for the Plackett-Burman DoE for each of the three responses.

Response	Metric			
	R^2	R^2 adjusted	Model p-value	Model fit equation
**Mist1**	0.76	0.60	0.004	$21.92 - 12.53 \times FGF10 + 20.35 \times EGFR\ inhibitor - 2.0 \times TGF\beta1\ inhibitor - 16.31 \times ROCK\ inhibitor - 0.51 \times Neurturin + 0.22 \times Apolipoprotein\ E - 2.47 \times Insulin - 19.84 \times EGFR\ inhibitor \times ROCK\ inhibitor + 14.20 \times Apolipoprotein\ E \times Insulin$
**Aqp5**	0.75	0.61	0.002	$2.17 + 0.70 \times FGF10 - 1.14 \times EGFR\ inhibitor - 0.08 \times TGF\beta1\ inhibitor + 1.14 \times ROCK\ inhibitor + 0.36 \times Neurturin + 0.03 \times Apolipoprotein\ E - 0.09 \times Insulin - 19.84 \times EGFR\ inhibitor \times ROCK\ inhibitor - 0.73 \times Apolipoprotein\ E \times Neurturin$
**Tmem16a**	0.86	0.75	0.0004	$26.49 + 10.30 \times FGF10 - 22.38 \times EGFR\ inhibitor - 14.90 \times TGF\beta1\ inhibitor - 10.42 \times ROCK\ inhibitor + 10.21 \times Neurturin - 6.60 \times Apolipoprotein\ E - 7.80 \times Insulin - 15.62 \times FGF10 \times Apolipoprotein\ E + 19.34 \times EGFR\ inhibitor \times TGF\beta R1\ inhibitor + 19.14 \times TGF\beta R1\ inhibitor \times ROCK\ inhibitor$

Table S4. ***P*-values for the individual factors and significant interactions for the Plackett-Burman DoE.** (-) indicates that the interaction was not included in the model for that response.

	Response		
**Factor/Interaction**	**Mist1**	**Aqp5**	**Tmem16a**
FGF10	0.034	0.031	0.044
EGFR inhibitor	0.002	0.001	0.000
TGFβR1 inhibitor	0.713	0.778	0.007
ROCK inhibitor	0.009	0.001	0.042
Neurturin	0.926	0.236	0.046
Apolipoprotein E	0.968	0.929	0.178
Insulin	0.651	0.770	0.117
EGFR inhibitor*ROCK inhibitor	0.004	-	-
Apolipoprotein E*Insulin	0.025	-	-
Neurturin*Apolipoprotein E	-	0.025	-
FGF10*Apolipoprotein E	-	-	0.011
EFGR inhibitor*TGFβR1 inhibitor	-	-	0.002
TGFβR1 inhibitor*ROCK inhibitor	-	-	0.002

Table S5. Relative mRNA expression results for Mist1, Aqp5, and Tmem16a for individual runs of the Box-Behnken DoE.

	Metric			
**Response**	**R^2**	**R^2 adjusted**	**Model *p*-value**	**Model fit equation**
**Mist1**	0.98	0.95	0.000	$12.00 + 7.17 \times EGFR\ inhibitor - 6.22 \times FGF10 + 2.27 \times Neurturin + 0 - 7.26 \times EGFR\ inhibitor \times FGF10 - 4.06 \times FGF10 \times Neurturin - 2.36 \times EGFR\ inhibitor \times EGFR\ inhibitor - 1.44 \times Neurturin \times Neurturin$
**Aqp5**	0.88	0.78	0.003	$1.56 - 2.95 \times EGFR\ inhibitor - 1.99 \times FGF10 - 0.93 \times Neurturin + 3.47 \times EGFR\ inhibitor \times FGF10 + 1.07 \times FGF10 \times Neurturin + 3.60 \times EGFR\ inhibitor \times EGFR\ inhibitor$
**Tmem16a**	0.94	0.88	0.001	$14.41 - 53.63 \times EGFR\ inhibitor - 12.28 \times FGF10 - 9.93 \times Neurturin + 21.19 \times FGF10 \times EGFR\ inhibitor + 52.47 \times EGFR\ inhibitor \times EGFR\ inhibitor - 19.00 \times FGF10 \times FGF10 + 14.57 \times Neurturin \times Neurturin$

Table S6. Model metrics (R^2, R^2 adjusted, model *p*-value) and model fit equation for the Box-Behnken DoE for each of the three responses.

Run #	Factor			Response		
	**EGFR inhibitor**	**FGF10**	**Neurturin**	**Mist1 (%)**	**Aqp5 (%)**	**Tmem16a (%)**
**1**	1	1	0	4.62	2.42	7.35
**2**	-1	0	-1	1.18	10.07	171.37
**3**	1	0	-1	11.90	4.70	29.04
**4**	-1	1	0	2.57	0.67	47.87
**5**	0	-1	1	24.34	0.50	11.16
**6**	0	0	0	11.25	1.03	5.58
**7**	0	0	0	5.76	3.38	23.48
**8**	1	-1	0	32.15	2.86	5.49
**9**	-1	0	1	2.16	6.70	123.15
**10**	0	-1	-1	9.56	3.01	17.36
**11**	0	1	1	4.38	2.06	6.58
**12**	0	0	0	16.54	0.20	14.15
**13**	0	1	-1	5.83	0.27	4.77
**14**	1	0	1	15.71	0.09	2.20
**15**	-1	-1	0	1.07	14.99	130.77

Table S7. *P*-values for the individual factors and interactions for the Box-Behnken DoE. (-) indicates that the interaction was not included in the model for that response.

Factor/Interaction	Response		
	**Mist1**	**Aqp5**	**Tmem16a**
EGFR inhibitor	0.000	0.003	0.000
FGF10	0.000	0.019	0.114
Neurturin	0.013	0.209	0.187
EGFR inhibitor*FGF10	0.000	0.007	0.063
FGF10*Neurturin	0.004	0.297	-
EGFR inhibitor*EGFR inhibitor	0.051	0.007	0.001
Neurturin*Neurturin	0.195	-	0.189
FGF10*FGF10	-	-	0.099

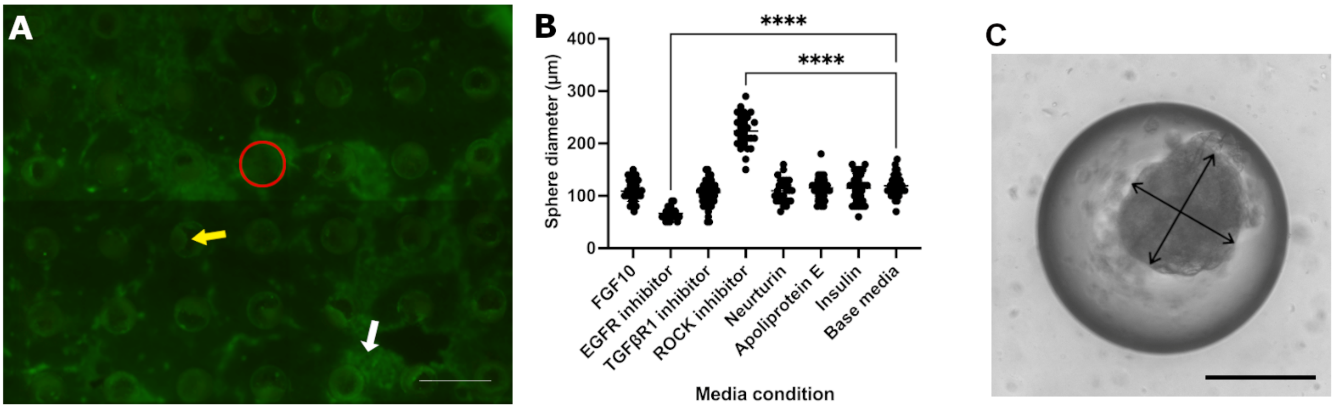

Figure S1. Morphological changes are induced by different media supplements. Live/dead staining of cells cultured with ROCK inhibitor Y-27632 for 7 days, showing extreme outgrowth of cells from the MBs (A). The red circle outlines an individual MB in the array. The yellow arrow points to a sphere growing inside a MB. The white arrow points to outgrowth cells on the surface of the chip. Scale bar is 600 µm. Sphere diameter quantification of tissue mimetics cultured for 7 days with each of the DoE factors individually (B). Statistical differences were analyzed with one-way ANOVA with comparison to base media. **** $p < 0.0001$. Example image of how sphere diameter was quantified using ImageJ (C). Two perpendicular lines were drawn across the sphere and measured using the scale set in ImageJ. Scale bar is 200 µm.

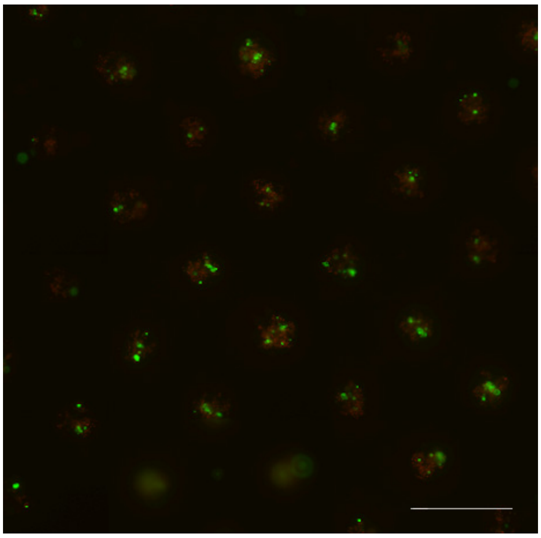

Figure S2. Higher concentrations of EGFR inhibitor show increased cytotoxicity and reduced sphere size. Live/dead staining of cells cultured with 1 µM EGFR inhibitor for 7 days (Figure 4.1B). Scale bar is 500 µm.

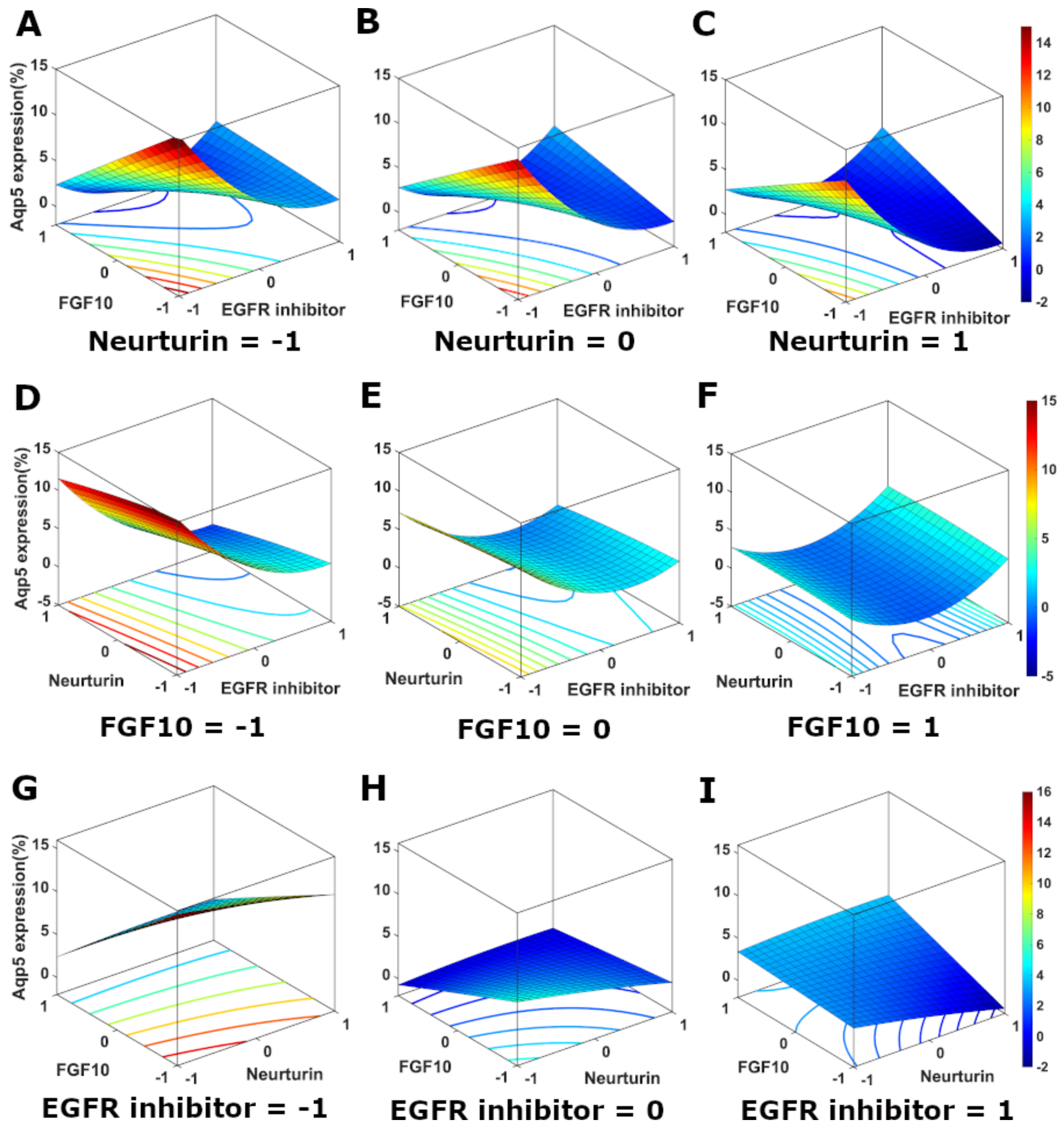

Figure S3. Optimal Aqp5 mRNA expression is predicted at low concentrations of FGF10 and neurturin. 3D surface response and contour plots for the Box-Behnken DoE for Aqp5 mRNA expression as a function of the concentration levels of EGFR inhibitor and FGF10 (A–C) with neurturin fixed at level -1 (A), 0 (B), and 1 (C); EGFR inhibitor and neurturin (D–F) at level -1 (D), 0 (E), and 1 (F); and neurturin and FGF10 (G–I) at level -1 (G), 0 (H), and 1 (I).

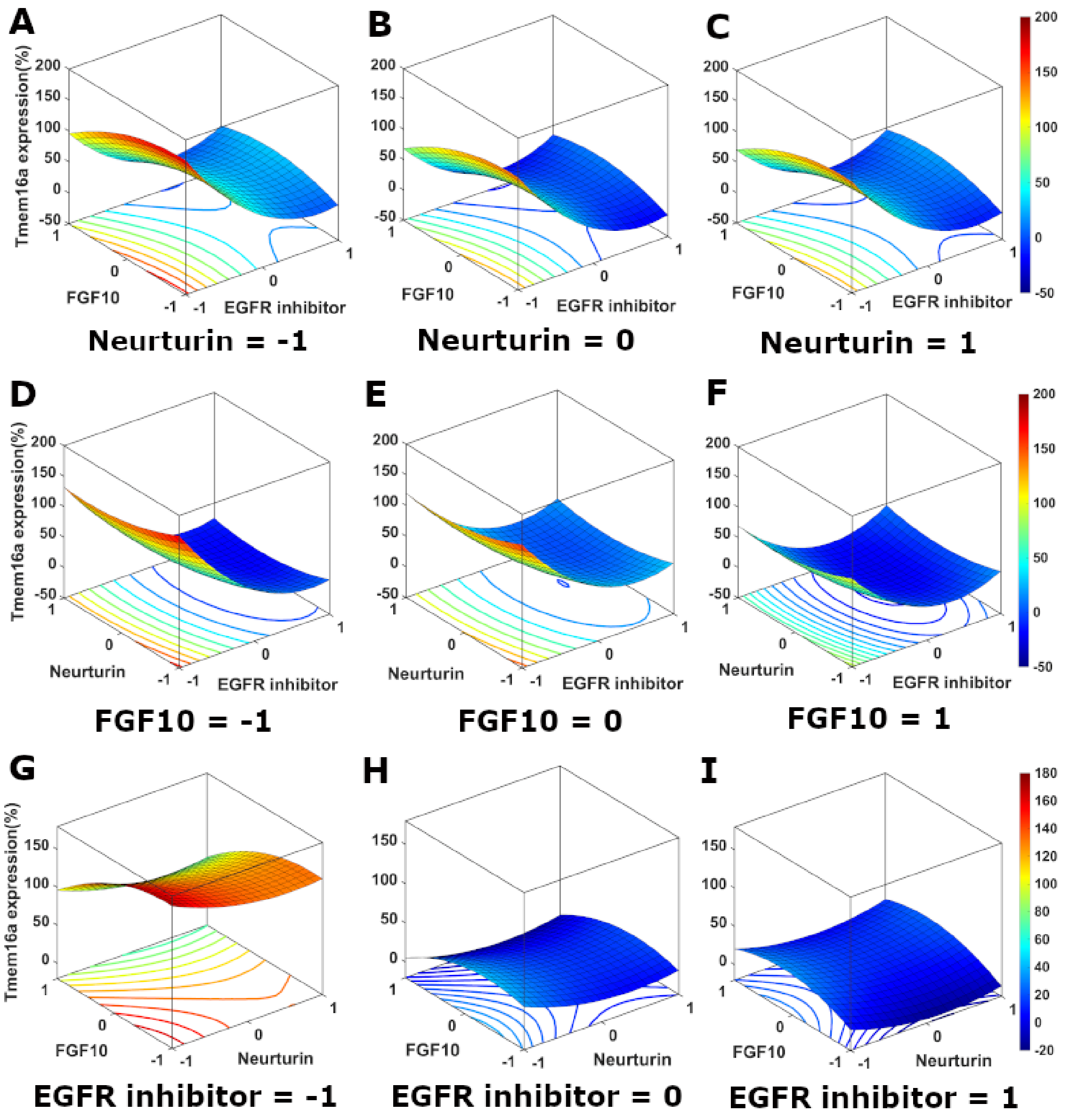

Figure S4. Optimal Tmem16a mRNA expression is predicted at low concentrations of FGF10 and neurturin. 3D surface response and contour plots for the Box-Behnken DoE for Tmem16a mRNA expression as a function of the concentration levels of EGFR inhibitor and FGF10 (A–C) with neurturin fixed at level -1 (A), 0 (B), and 1 (C); EGFR inhibitor and neurturin (D–F) at level -1 (D), 0 (E), and 1 (F); and neurturin and FGF10 (G–I) at level -1 (G), 0 (H), and 1 (I).

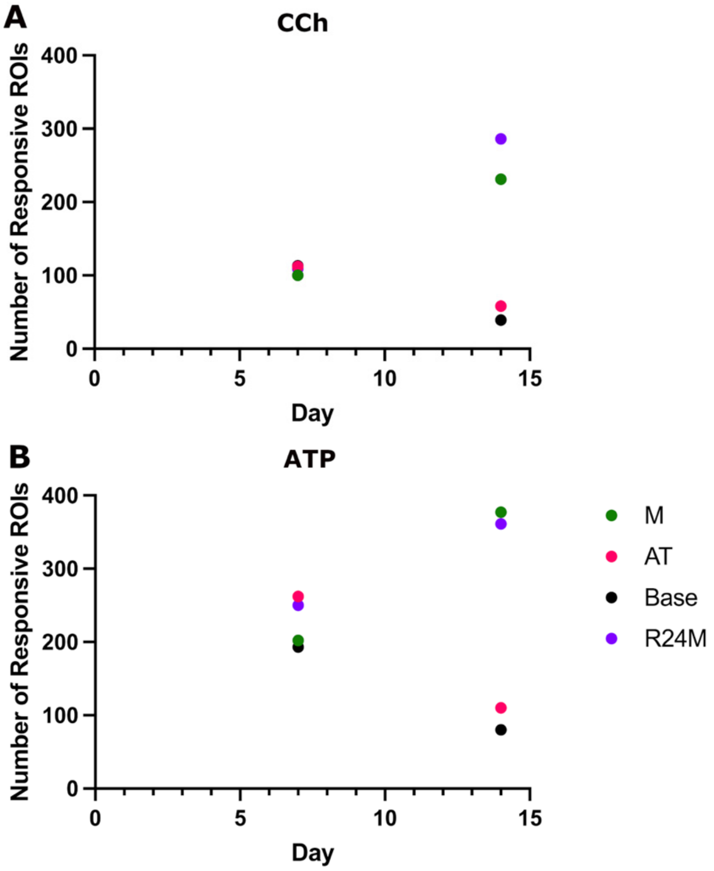

Figure S5. M and R24M media show increased number of responsive ROIs at day 14. Number of responsive ROIs that respond to CCh (A) or ATP (B) at days 7 and 14.

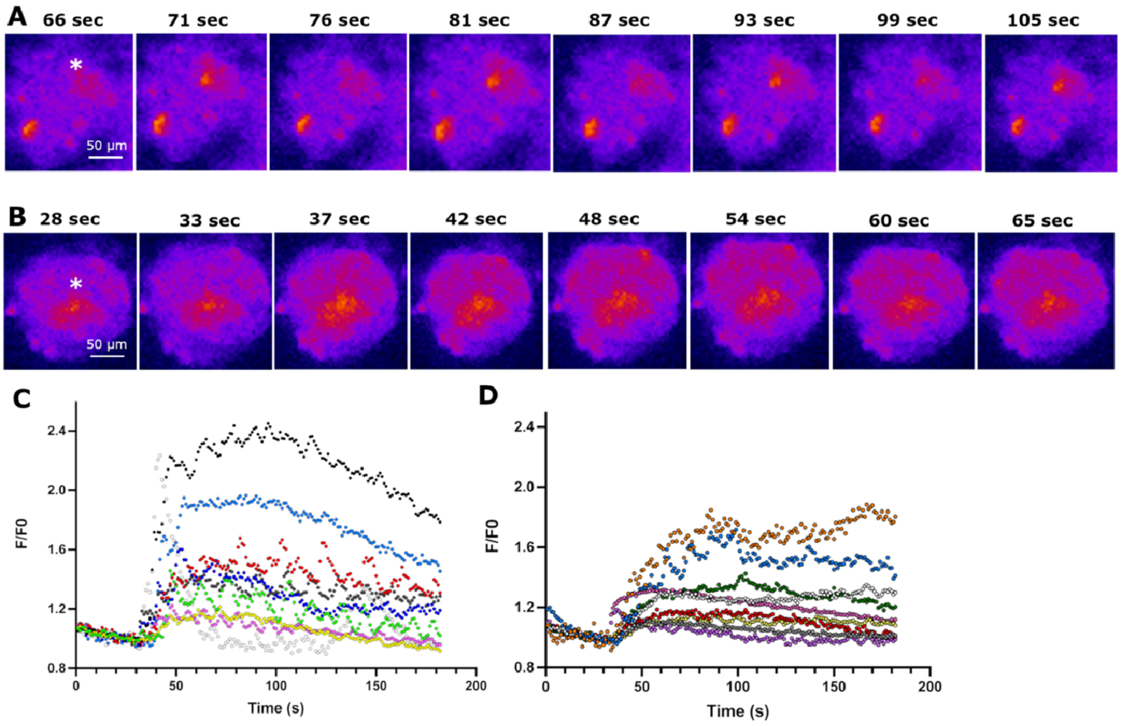

Figure S6. Calcium signaling traces of individual ROIs showing oscillatory behavior in M media. Time series images of a single tissue mimetic in M media (A). Area of interest in marked with an *. Time series images of a single tissue mimetic in base media (B). Area of interest in marked with an *. A selection of calcium signaling traces from individual ROIs in M (C) or base (D) media.

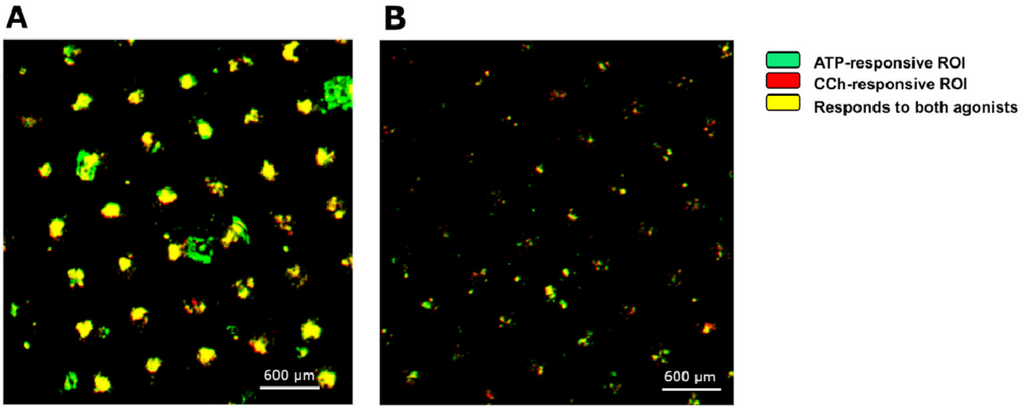

Figure S7. Distinct regions respond to CCh vs. ATP in M media. Overlap between CCh- (red) and ATP-responsive (green) ROIs with overlap shown in yellow for base (A) and M (B) media.

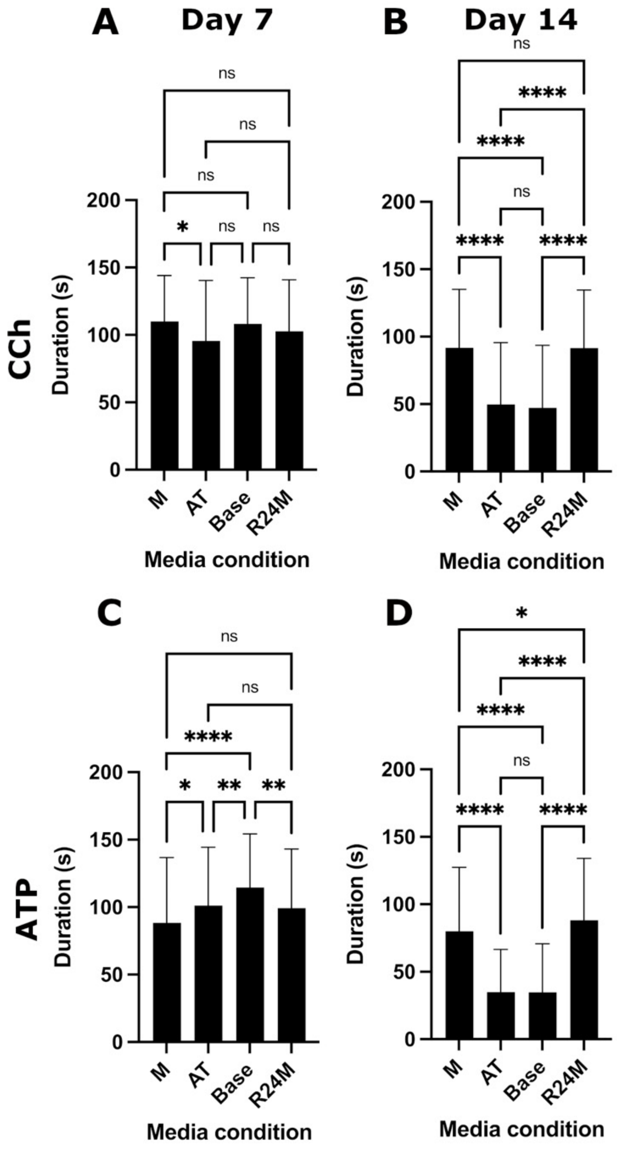

Figure S8. M and R24M media show increased duration of the calcium signaling response at day 14. Duration of the calcium signaling response at day 7 (A,C) and day 14 (B,D) for CCh (A,B) and ATP (C,D). Data is represented as mean ± SD (n = 3). Statistics were calculated using one-way ANOVA with Tukey's multiple comparisons. ns = nonsignificant, * $p < 0.05$, ** $p < 0.01$, **** $p < 0.0001$.

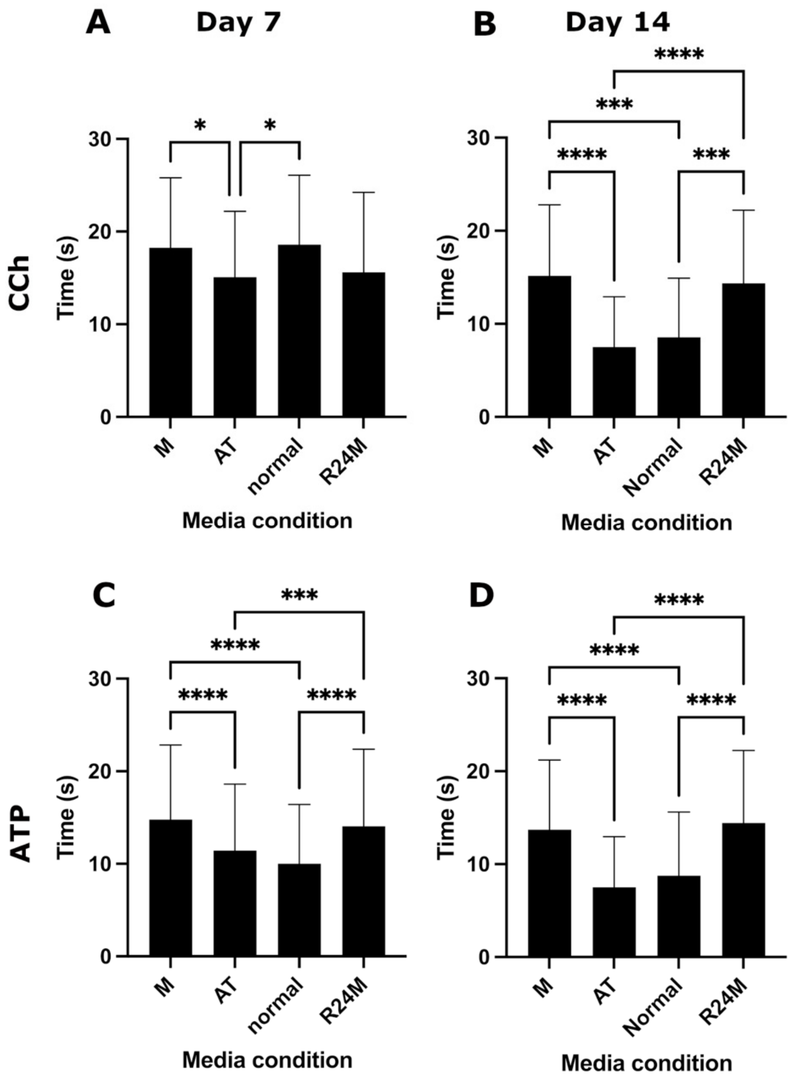

Figure S9. M and R24M media show longer latency of the calcium signaling response at day 14. Latency of the calcium signaling response at day 7 (A,C) and day 14 (B,D) for CCh (A,B) and ATP (C,D). Data is represented as mean ± SD (n = 3). Statistics were calculated using one-way ANOVA with Tukey's multiple comparisons. ns = nonsignificant, * $p < 0.05$, ** $p < 0.01$, *** $p < 0.001$, **** $p < 0.0001$.

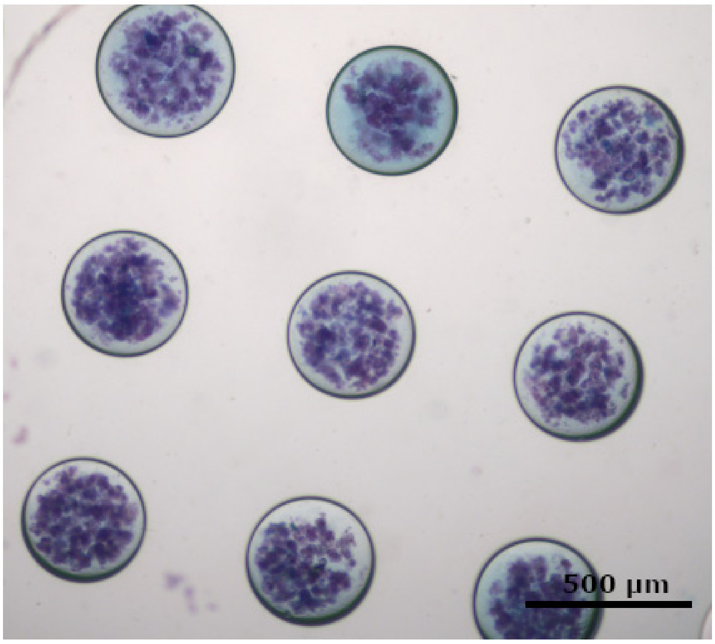

Figure S10. Freshly isolated salivary gland cells in MBs show expression of both acidic and neutral mucins using PAS-AB staining.
